# High prevalence of hepatitis B virus genotype C/C1 in the Minangkabau ethnic group in Indonesia

**DOI:** 10.1186/1743-422X-10-27

**Published:** 2013-01-22

**Authors:** Marlinang D Siburian, Andi Utama, Rama Dhenni, Arnelis, Ismail Fanany, Mariana DB Intan, Tri S Kurniasih, Febi Andriani, Szeifoul Afadlal, Erlys B Julianto, Widyarman S Rasman, Nasrul Zubir, George Mathew

**Affiliations:** 1 Molecular Epidemiology Division, Mochtar Riady Institute for Nanotechnology, Universitas Pelita Harapan, Lippo Karawaci, Tangerang, Indonesia; 2 Department of Internal Medicine, M. Djamil Hospital, Padang, Indonesia; 3 Red Cross Unit, Padang, Indonesia

**Keywords:** HBV genotype, Pre-S mutation, BCP mutation, Minangkabau

## Abstract

**Background:**

The Minangkabau is one of the major ethnic groups in Indonesia. Previous studies with a limited number of samples have shown a different prevalence of HBV/C in the Minangkabau compared to the Indonesian population in general. The aim of this study was to assess the HBV genotype distribution pattern and the prevalence of pre-S, T1753V and A1762T/G1764A mutations among the Minangkabau HBV carriers. The samples were collected from Padang, West Sumatera and from western Java. Mixed primers for specific genotypes were used to determine the HBV genotype. Pre-S or S genes were amplified, sequenced and aligned with reference sequences from GenBank to derive a phylogenetic tree for subgenotyping. Pre-S genes were also analyzed for mutations. The basal core promoter (BCP) region was amplified and directly sequenced to analyze T1753V and A1762T/G1764A mutations.

**Results:**

The predominant HBV genotype among the Minangkabau HBV carriers (n=117) was C (72.6%) followed by B (24.8%) and co-infection with B and C (2.6%). The prevalence of pre-S mutations, including both the pre-S deletion and pre-S2 start codon mutation, was 41.0%, and the T1753V and A1762T/G1764A mutations were found in 51.9% and 71.2% respectively. HBV/C1 was the predominant HBV subgenotype in the Minangkabau HBV carriers, and was found in 66.2%, followed by B3, B7, C8, B2, B9, C2, and C10 (18.3%, 7.0%, 2.8%, 1.4%, 1.4%, 1.4%, and 1.4% respectively). From samples that were found to be co-infected with HBV B and C, two samples were successfully cloned and subgenotyped, including one with mixed subgenotypes of B3 and C1, and another one with mixed subgenotypes of B7, C1, putative intergenotypic of B/A, and C/A. Furthermore, three samples from donors of non-Minangkabau ethnicity from Padang were found to be infected with an intragenotypic recombination form, including a putative recombinant of B8/B3 and B9/B7.

**Conclusion:**

HBV/C with subgenotype C1 was the predominant HBV genotype among HBV carriers of Minangkabau ethnicity. The prevalence of pre-S, A1762T/G1764A, and T1753V mutations was higher among the Minangkabau compared to Indonesian HBV carriers in general.

## Background

Hepatitis B virus is still one of the highest burden diseases in the world. It is estimated that 30% of the world’s population has had contact with or are carriers of the hepatitis B virus (HBV) [1]. More than 75% of the 350 millions of HBV carriers are located in Western Pacific and South East Asia region [2]. Although the incidence of acute HBV infection has decreased in most countries due to the implementation of vaccination programs, HBV-related complications such as cirrhosis and hepatocellular carcinoma (HCC) are still increasing [1]. HBV is responsible for chronic hepatitis progressing to cirrhosis (10–20%), and as much as 20–30% of compensated cirrhosis will lead to hepatic decompensation, and 5–15% of compensated cirrhosis will lead to HCC [3].

Classification of the HBV genotype was based on >8% intergenotype and <4% intragenotype divergences [4,5]. Ten HBV genotypes (A-J) have been identified [6,7], and each genotype can be further classified into subgenotypes [4]. HBV genotypes have extremely uneven geographic distributions, which can help in tracing its migration [5]. Genotypes B and C account for more than 90% of chronic HBV infection in East Asia [8]. HBV genotype B is associated with spontaneous HBeAg seroconversion at a younger age, with less active liver disease, and a slower rate of progression to cirrhosis compared with HBV genotype C [8,9]. Furthermore, HBV genotype C and specific mutations of the basal core promoter (BCP) and precore regions were associated with risk of HCC independent of serum HBV DNA level [10].

Indonesia has moderate to high endemicity for HBV infection, with a carrier rate of 5–20% in the general population [11]. Genotype B is the predominant HBV genotype in Indonesia, followed by genotypes C, D, and A [12-14]. HBV/B is predominant in the western part of Indonesia, whilst HBV/C is dominant on the eastern part [13,14]. Mutations at the BCP region such as A1762T/G1764A and T1753V were found in high prevalence (59.5% and 40.5%, respectively) and were associated with severe liver disease [12]. In a more recent study, the prevalence of pre-S mutation in Indonesian population was also reported [15,16].

The Minangkabau ethnic group is one of the seven major ethnic groups in Indonesia. They originated from and still mainly reside in the West Sumatra province of Sumatra Island [17]. Previous studies have shown that HBV genotype C is the most prevalent among HBV carriers in this population, which is different from other populations in Sumatra Island [12-14]. However, the numbers of subjects in these studies were low. The aims of the present study were to assess the HBV genotype distribution as well as to determine the prevalence of mutations in the pre-S and BCP regions of the HBV genome in HBV carriers of the Minangkabau ethnic group. We investigated those of Minangkabau ethnicity who reside in Padang, the capital city of West Sumatra province, and those in western Java. In addition, we compared the genotype distribution among those of Minangkabau with those of Javanese ethnicity, the largest ethnic group in Indonesia.

## Results

### Demographic data of subjects in Padang population

A total of 189 HBsAg positive blood donors and liver disease patients from the Padang region were included in the study. The ethnicities of the 189 were Minangkabau (59.3%), half Minangkabau (only mother or father is of Minangkabau ethnicity) (19.6%), Javanese (7.4%), Bengkulu (2.1%), Nias (2.1%), Batak (1.6%), Sundanese (1.6%), Jambi (1.1%), Kerinci (1.1%), Mentawai (1.1%), and others (3.2%). From the 189 samples, 140 samples were successfully genotyped and were grouped into the Minangkabau and the non-Minangkabau ethnic group (Table 1). The mean ± SD age of the 140 samples was 37.5 ± 11.6 years old and about 87.1% of them were male. There was no significant difference in mean age and gender between the two groups. Of the samples, 75.7% (106/140) were from blood donors and 24.3% (34/140) were from patients with liver disease. HBV genotypes found in Padang population were B (35.7%), C (60.7%), and co-infection of B and C (3.6%). The prevalence of HBV/C in the Minangkabau ethnic group was higher than in the non-Minangkabau ethnic group (75.9% vs 38.6%, *P*<0.0003). Pre-S mutations in samples from Padang were found in the form of pre-S deletion and pre-S2 start codon mutations. From 140 samples genotyped, 79 samples were successfully analyzed for pre-S mutation. Thirty two of 79 samples (40.5%) had pre-S mutations, including seven (8.9%) with both pre-S deletion and mutation at the pre-S2 start codon, twelve with pre-S deletion alone (15.2%) and thirteen (16.5%) with pre-S2 start codon mutations only. Mutations at the BCP region could only be analyzed in 58 of the 140 genotyped samples. The mutations A1762T/G1764A and T1753V were found in high prevalence, 35/58 (60.3%) and 21/58 (36.2%) respectively. Mutations at both the pre-S and BCP regions were found more frequently in the Minangkabau than in the non-Minangkabau ethnic groups. Furthermore, the prevalence of the T1753V mutation in the Minangkabau ethnic group was significantly higher than in the non-Minangkabau ethnic group (50.0% vs 19.2%, *P*=0.015) (Table 1).

**Table 1:** Demographic data and HBV variants mutant prevalence of HBV carrier in Padang population

	**Minangkabau, n=83**	**Non-Minangkabau, n=57**	**All, n=140**	***P***
Male/Female, (%male)	72/11 (86.7)	50/7 (87.7)	122/18 (87.1)	0.866
Age ± SD	38.6±12.1	35.8±10.6	37.5±11.6	0.156
AC:CH:LC:HCC	55:8:17:3	51:0:5:1	106:8:22:4	0.010
Genotype, n(%)				
B	17 (20.5)	33 (57.9)	50 (35.7)	2.76E-05
C	63 (75.9)	22 (38.6)	85 (60.7)	
B/C	3 (3.6)	2 (3.5)	5 (3.6)	
Pre-S mutation*	25 (50.0)	7 (24.1)	32 (40.5)	0.024
Pre-S deletion*	14 (28.0)	5 (17.2)	19 (24.1)	0.281
Pre-S2 start codon mut*	16 (32.0)	4 (13.8)	20 (25.3)	0.073
A1762T/G1764A**	22 (68.8)	13 (50.0)	35 (60.3)	0.147
T1753V**	16 (50.0)	5 (19.2)	21 (36.2)	0.015

*AC* Asymptomatic carrier, *CH* Chronic hepatitis, *LC* Liver cirrhosis, *HCC* Hepatocellular carcinoma.

*All=79, Minangkabau=50, Other=29.

**All=58, Minangkabau=32, Other=26.

### Demographic data of Minangkabau and Javanese ethnic groups

A total of 117 plasma samples from HBV carriers of Minangkabau ethnicity (83 from Padang and 34 from western Java areas) were compared with 92 plasma samples of Javanese HBV carriers (9 from Padang and 83 from western Java areas) as shown in Table 2. The mean ± SD age of the Minangkabau ethnic group was younger than the Javanese (40.9 ± 13.0 vs 45.0 ± 12.1 years old, *P*=0.020), while the ratios of male/female between the two groups were not significantly different. Samples in the Javanese ethnic group were mostly from liver disease patients (91.3%), whilst almost half (47.0%) of samples from Minangkabau ethnic group were from blood donors (Table 2). The predominant genotype in the Minangkabau ethnic group was C (73.5%) followed by B (23.9%) and co-infection of B and C (2.6%). Conversely, in the Javanese ethnic group the predominant genotype was B (88.0%) followed by C (10.9%), and co-infection of B and C (1.1%) (Table 2).

**Table 2:** Demographic data and prevalence of HBV mutant variants of HBV carrier in Minangkabau and Javanese ethnics

	**Minangkabau, n=117**	**Javanese, n=92**	***P***
Male/Female, (%male)	93/24 (79.5)	67/25 (72.8)	0.259
Age ± SD	40.9±13.0	45.0±12.1	0.020
AC:CH:LC:HCC	55:28:26:8	8:38:29:17	3.55618E-08
Genotype, n(%)			
B	28 (23.9)	81 (88.0)	3.21039E-19
C	86 (73.5)	10 (10.9)	
B/C	3 (2.6)	1 (1.1)	
Pre-S mutation**	25 (41.0)	20 (45.5)	0.648
Pre-S deletion**	15 (24.6)	5 (11.4)	0.089
Pre-S2 start codon mut**	15 (24.6)	18 (40.9)	0.076
A1762T/G1764A***	37 (71.2)	26 (41.9)	0.002
T1753V***	27 (51.9)	17 (27.4)	0.007

*AC* Asymptomatic carrier, *CH* Chronic hepatitis, *LC* Liver cirrhosis, *HCC* Hepatocellular carcinoma.

**All=105, Minangkabau=61, Javanese=44.

***All=114, Minangkabau=52, Javanese=62.

The prevalence of HBV mutations between ethnic groups showed different distribution patterns. For pre-S mutations analysis 61 of 117 samples from the Minangkabau ethnic group and 44 of 92 samples from the Javanese ethnic group were successfully amplified. Pre-S mutations were found more prevalent in the Javanese than in the Minangkabau ethnic group (45.5% vs 41.0%). The pre-S2 start codon mutation was more common than pre-S deletion (34.1% vs 4.5%, respectively) in the Javanese ethnic group, whilst both type of mutations showed similar prevalence (16.4%) in the Minangkabau ethnic group. Conversely with the pre-S mutation, mutations at BCP region were more prevalent in the Minangkabau ethnic group than in the Javanese ethnic group. For the BCP mutation analysis, 52 of 117 samples from the Minangkabau ethnic group and 62 of 92 samples from the Javanese ethnic group were successfully amplified. The T1753V mutation was found in 51.9% and 27.4% of the Minangkabau and Javanese ethnic groups (*P*=0.007), whilst the A1762T/G1764A mutation was detected in 71.2% and 41.9% of the Minangkabau and Javanese ethnic groups (*P*=0.002) (Table 2).

### HBV subgenotype analysis

**Figure 1:**
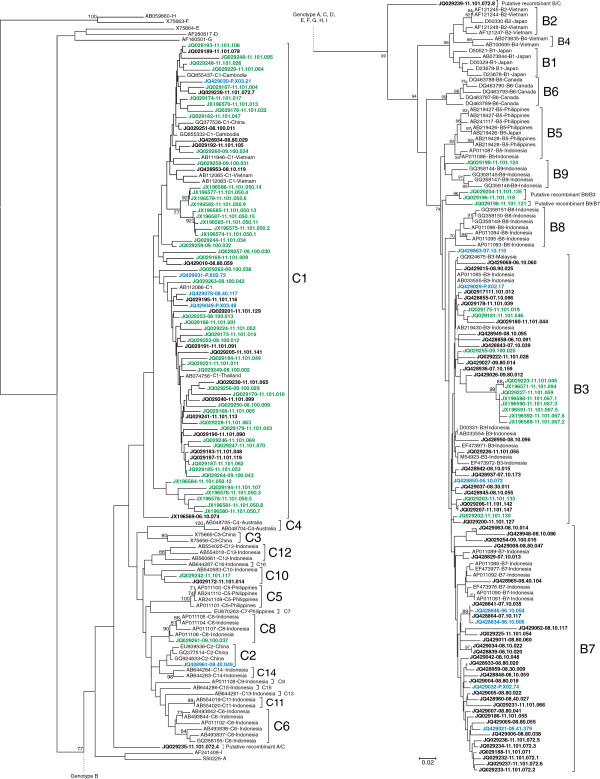
**The Neighbor-joining phylogenetic tree of the 436 nt pre-S sequence of 132 reported HBV strains from Padang and West Java obtained in the present study (indicated with the accession numbers and sample ID) together with all reference sequences from GenBank (indicated with the accession numbers, genotypes/subgenotypes, and country).** HBV sequences typed in bold black are samples from non-Minang ethnic in Padang and West Java, in bold green are samples from Minang ethnic in Padang, and in bold blue are samples from Minang ethnic in western Java. The HBV genotypes/subgenotypes are indicated on the right of each respective cluster. Bootstrap values higher than 70% are shown at major branches. The length of the horizontal bar indicates the number of nucleotide substitution per site.

**Figure 2:**
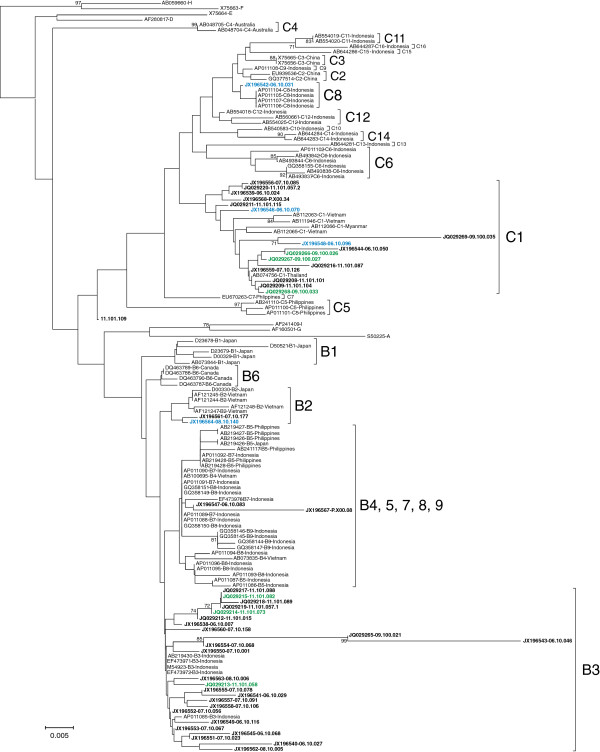
**The Neighbor-joining phylogenetic tree of the 323 nt S sequence of 45 reported HBV strains from Padang and West Java obtained in the present study (indicated with the accession numbers and sample ID) together with all reference sequences from GenBank (indicated with the accession numbers, genotypes/subgenotypes, and country).** HBV sequences typed in bold black are samples from non-Minang ethnic in Padang and West Java, in bold green are samples from Minang ethnic in Padang, and in bold blue are samples from Minang ethnic in western Java. HBV genotypes/subgenotypes are indicated on the right of each respective cluster. Bootstrap values higher than 70% are shown at major branches. The length of the horizontal bar indicates the number of nucleotide substitution per site.

**Figure 3:**
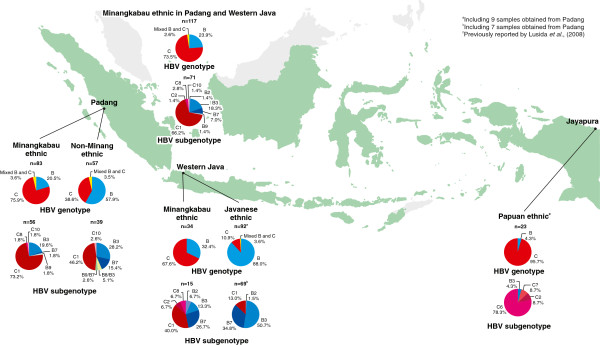
**Distribution of HBV genotypes/subgenotypes obtained in this study from Padang and western Java (Jakarta and Tangerang) in comparison with HBV genotypes/subgenotypes from Papua, previously reported by Lusida et al.**[22]**.**

A total of 172 plasma samples (56 and 39 samples from the Minangkabau and the non-Minangkabau ethnic groups in Padang, with the addition of 15 and 62 samples with Minangkabau and Javanese ethnicity from previous study in western Java) were successfully subgenotyped. Phylogenetic trees were constructed based on the pre-S sequence (n=129) or the S gene (n=43) (Figures 1 and 2). In the Padang population, the predominant subgenotype was C1 followed by B3, B7, C10, B9, and C8 (Figure 3). The prevalence of HBV/C1 in the Minangkabau group was significantly higher than in the non-Minangkabau ethnic group, although HBV/C1 was predominant in both groups (73.2 vs 46.2, *P*=0.014). In samples with co-infection of HBV/B and C, the subgenotype could only be determined in two samples, both of the Minangkabau ethnic group (11.101.057 and 11.101.072). From sample 11.101.057, five clones of the S gene were successfully retrieved, including four clones of HBV/B3 and one clone of HBV/C1. From sample 11.101.072 eight clones of the pre-S gene were retrieved, including five clones with HBV/B7, one HBV/C1, one with putative intergenotypic recombinant of B/A and the last one with putative intergenotypic recombinant of C/A. Furthermore, three samples were found to be infected with HBV intergenotypic recombinant (11.101.118, 11.101.121, 11.101.135). All three were found in the non-Minangkabau ethnic group. Samples 11.101.118 and 11.101.135 were found to be infected with an HBV putative recombinant of B8/B3, and 11.101.121 was found with a putative recombinant of B9/B7.

Eighty three samples from HBV carriers of Minangkabau ethnicity collected from the Padang population were compared to 34 samples from HBV carriers of Minangkabau ethnicity that resided in western Java. The HBV genotype distribution patterns of the two groups were found to be similar (Figure 3). Genotype C was the predominant genotype in the Minangkabau ethnic group from both Padang and western Java (75.9% vs 67.6%). Despite the similar genotype distribution pattern, the subgenotype distribution in the groups was different, although the predominant subgenotype was the same (HBV/C1). The subgenotypes found in the Minangkabau ethnic group in Padang were C1, B3, B7, B9, C8, and C10 (73.2%, 19.6%, 1.8%, 1.8%, 1.8%, and 1.8% respectively). Whilst the subgenotypes found in the Minangkabau ethnic group in western Java were C1, B7, B3, B2, C2, and C8 (40.0%, 26.7%, 13.3%, 6.7%, 6.7%, and 6.7% respectively).

The HBV genotype and subgenotype distributions were significantly different between Minangkabau and Javanese HBV carriers (Figure 3). The subgenotypes found in the Minangkabau ethnic group (from Padang and western Java) were: C1, B3, B7, C8, B2, B9, C2, and C10 (66.2%, 18.3%, 7.0%, 2.8%, 1.4%, 1.4%, 1.4% and 1.4% respectively), whereas four subgenotypes were observed in the Javanese ethnic group including B3, B7, C1, and B2 (50.7%, 34.8%, 13.0% and 1.5% respectively).

## Discussion

Particular HBV genotypes are associated with the outcome of chronic HBV infection and the response to antiviral therapies [8,18-20]. The prevalence of HBV genotypes varies geographically, where genotypes B and C account for more than 90% of chronic HBV infection in East Asia [8]. Furthermore, HBV strains circulating in an area can reflect the ethnic mix of its population [13,14,21]. In the western part of Indonesia (Sumatra, Nias, Mentawai, Kalimantan, Java and Lombok islands), HBV/B was predominant [13]. Interestingly, in Minangkabau population of West Sumatra the predominant HBV genotype is HBV/C with subgenotype C1 [13,14]. HBV/C was found to be predominant in the eastern part of Indonesia (Papua and Papua-influenced populations of Moluccas) but of different subgenotypes than those found in the western Indonesia (C6 and C7) [13,14,22].

HBV/C was highly prevalent in the Minangkabau ethnic group, whilst in non-Minangkabau ethnic groups HBV/B was more prevalent. In the western Indonesian population, the HBV/C was exclusively high in Minangkabau population [13]. While our data supports the data from previous studies, it also reveals the distinct HBV genotypes that are conserved in those of Minangkabau ethnicity residing in Padang, as well as those of who live in western Java (Figure 3). Minangkabau ethnic people have strong traditions, and usually marry within their own group. Studies of Minangkabau associations in different regions indicate that they often develop out of pre-existing kin or locality-based networks that promote social interactions among their members [23]. The present data illustrate a conserved transmission route of HBV infection which was most likely vertical, as it was obtained in high prevalence areas where HBV infection usually occurs perinatally or during infancy and early childhood [18]. Thus, distinct HBV genotypes can be kept in societies living closely together even after migration over long distances [24].

The HBV genotype C is independently associated with a higher risk of HCC and associated with more rapid progression to cirrhosis than genotype B [8,20,23]. A prospective study from Hong Kong of 1,006 patients with chronic HBV infection followed up for a median of 7.7 years showed that the highest risk of developing HCC was in persons infected with HBV genotype C2, with the next highest being C1, followed by those infected with genotype B (presumably Ba) [20]. Differences between the Asian genotypes B and C appears to be influenced by the subgroup of genotype B, because in Japan where the genotype B1 (Bj) is prevalent, no differences in the development of HCC between genotypes B and C was observed [24]. The samples in this study were mostly from blood donors, however the data demonstrated a higher proportion of patients with liver disease among those of Minangkabau ethnicity than in the non-Minangkabau ethnic group (*P*=0.010) (Table 1).

Different HBV genotypes display distinct patterns of mutations at the Pre-S region and at the EnhII, BCP, and precore (EnhII/BCP/Precore) region in the HBV genome [10]. The prevalence of pre-S mutants among different HBV genotypes was significantly higher in patients with genotypes B (25.0%) and C (24.5%) than the other genotypes (*P*<0.05), but there was no significant difference between genotypes B and C [25]. The A1762T/G1764A mutation is more commonly found in patients with genotype C than those with genotype B [8]. In Padang population, the data demonstrated significantly higher prevalence of pre-S mutant, T1753V, and A1762T/G1764A in the Minangkabau compared to the non-Minangkabau ethnic group. This was due to the higher prevalence of HBV/C and liver disease patients who provided samples from the Minangkabau ethnic group. The pre-S mutation was mostly found in HBV/C and in samples from liver disease patients of the Padang population (data not shown).

In contrast to the results in Padang population, pre-S mutations in samples from the Minangkabau ethnic group (from Padang and western Java) were less prevalent compared to those from the Javanese ethnic group, although this difference was not statistically significant. In particular, pre-S2 start codon mutation was more common in samples from the Javanese ethnic group than in those from the Minangkabau ethnic group (Table 2). This is most likely due to the higher percentage of liver disease patients in the Javanese ethnic group. The frequencies of mutations at the pre-S2 promoters were significantly higher in the patients with HCC than in the patients without HCC (pre-S2 promoter mutation: 15.3% vs 8.9%, P=0.032) [10]. Furthermore, pre-S2 start codon mutation was associated with advanced liver disease and was the more common type of pre-S mutation in Indonesian patients regardless of the HBV genotype [15,16].

The prevalence of the A1762T/G1764A mutation was significantly higher in the Minangkabau compared to the Javanese ethnic group. This result demonstrated the high prevalence of the A1762T/G1764A mutation in HBV/C regardless of the stage of infection. A previous meta-analysis study, evaluating 43 studies with a total of 11582 HBV-infected participants, found that the frequency of C1653T, T1753V, and TA mutations increased successively from asymptomatic carrier to cirrhosis, and were independent factors associated with HCC. The C1653T mutation in HBV subgenotype C2 and T1753V and A1762T/G1764A in HBV subgenotypes C1 and C2 were statistically significantly associated with an increased risk of HCC. Furthermore, the study mentioned that pre-S mutations C1653T, T1753V, and A1762T/G1764A accumulated during the progression of chronic HBV infection from the asymptomatic carrier state to HCC (*P*_*trend*_<0.001 for each mutation) [10].

Random errors and variations in the HBV genome that occur over long periods while immune selection pressures operate at the population level have led to the emergence of distinct genotypes and subgenotypes in specific geo-ethnic populations. Since these variants are transmission competent, they can stably circulate within the given geo-ethnic population [26]. HBV genotypes may add additional support to anthropological data on ancient migration events [24]. The distribution of HBV genotypes/subgenotypes in the Indonesian archipelago is related to the ethnic origin of its populations and suggests that the HBV distribution is associated with the ancient migratory events in the peopling of the archipelago [13].

HBV/C1 was commonly found in Southeast Asia (the western part of Indonesia, Malaysia, Thailand, Vietnam and Bangladesh) and Southern China [12,13,20,21,27]. The Malay populations in the western (Melayu Minangkabau) and southern parts (Melayu Jawa and Melayu Bugis) of the Peninsular Malaysia were believed to have had more historical and cultural links with the populations from the Indonesian archipelago [28]. The Neighbor-Joining tree based on the genetic distance measure of Fst (a method to show population genetic structure by partitioning genetic variance within populations relative to between populations), demonstrated that the Malay Minangkabau are grouped with the Indonesian Melayu. This topology may reflect the migrations of Malay Minangkabau to Malay Peninsula from Sumatra, the geographic origins of Indonesian Melayu [28]. Genotypes B and C were equally frequent in ethnic Malays, where most genotype C strains were subgenotype C1 [21].

The multiethnic origin of the populations is reflected in the HBV genotype distribution in different parts of the country [26]. The successfully subgenotyped samples from the non-Minangkabau ethnic group classified in this study was comprised of half Minangkabau descendants, Javanese and Sundanese (originating from Java island), and ethnic groups from other parts of Sumatra island (41.0%, 23.1%, 35.9%, respectively). Most of the HBV/C1 samples from the non-Minangkabau ethnic group belong to the half Minangkabau (72.2%) and Bengkulu people (11.1%). Bengkulu is a province near West Sumatera province. On the other hand, most of the HBV/B3 and HBV/B7 were found in the Javanese (45.5% and 66.7%, respectively). Three samples with intragenotypic recombinant HBV were detected in the non-Minangkabau ethnic group, two with B8/B3 and one with B9/B7. The samples that contained B8/B3 recombinant were detected in blood donors originated from Mentawai (11.101.135), an island near West Sumatra province, and from Simeuleu (11.101.118), an island near Aceh province, whilst the B9/B7 genotype was detected in a blood donor sample that originated from Bengkulu (11.101.121). Co-infection with more than one HBV genotype may also be a more common occurrence than what was previously thought. Among the Asian patients from Vietnam, significantly higher associations were found between mixed genotype infections and acute hepatitis B, liver cirrhosis, and HCC although the specific combination was impossible to identify due to the different mixtures reported [20]. There were five samples from Padang population (1.9%) that were found to be co-infected with HBV/B and HBV/C, and all five were from blood donors. Two out of five were successfully cloned for subgenotyping (11.101.057 and 11.101.072). From sample 11.101.057, the S region was amplified and cloned. Five clones were obtained, four clones were HBV/B3 and one was HBV/C1. Whilst from sample 11.101.072, the pre-S region was successfully amplified and cloned. Interestingly, a mixture of subgenotypes variants was found. From eight clones obtained, five clones were HBV/B7, one clone was a putative intergenotypic recombinant strain of B/A, one clone was HBV/C1, and one clone was a putative intergenotypic recombinant strain of C/A.

In conclusion, the prevalence of HBV in blood donors in Padang was relatively low. The predominant HBV genotype and subgenotype in HBV carriers of Minangkabau ethnic group is HBV/C with subgenotype C1, which is different from the Indonesian population in general. The transmission route of HBV infection is most likely vertical, which allowed circulation of a conserved HBV genotype within the Minangkabau ethnic population. The prevalence of pre-S, A1762T/G1764A, and T1753V mutations were higher among HBV carriers of Minangkabau ethnicity. However, the association of the high prevalence of HBV/C1 and mutant variants with increased risk of advance liver disease among HBV carriers of Minangkabau ethnicity needs further investigation.

## Methods

### Samples

HBsAg screening tests were done for plasma samples from blood donors at the Padang Red Cross Unit from June 2010-July 2011. From 27,557 blood donor samples, 147 samples (0.53%) were found positive for HBsAg. In addition to the 147 HBsAg-positive samples from blood donors, 42 samples collected from HBV-related liver disease patients at the M. Djamil Hospital Padang were also included and made a total of 189 samples analyzed for genotyping. The liver disease samples comprised of 16 samples from patients with chronic hepatitis (CH), 22 samples from those with liver cirrhosis (LC), and 4 samples from those with HCC. Chronic hepatitis patients were defined as those positive for HBsAg for more than 6 months, and have more than twice the normal ALT level. Liver cirrhosis was diagnosed by liver function tests and ultrasonography, whilst the diagnosis of HCC was either on the basis of ultrasonography as well as an elevated serum α-fetoprotein (AFP) level (≥ 200 ng ml^-1^), or by needle aspiration liver biopsy for samples in which the AFP level was low. From 189 samples, 140 samples were successfully genotyped. The 140 samples were grouped into Minangkabau ethnicity and other ethnicity. HBsAg test for liver disease samples were performed using a commercially available ELISA kit (Abbott Laboratories, Chicago, IL), whilst HBsAg test for samples from blood donors were done by ELISA kit Hepanostika® HBsAg Ultra (BioMérieux SA, Marcy l'Etoile, France). Blood samples were collected from subjects at the time of their clinical evaluation or blood donation, separated into plasma and stored at −70°C until use. The study was approved by the Committee on Health Research Ethics of the Mochtar Riady Institute for Nanotechnology and informed consent was obtained from each subject.

For comparison purpose, we included 117 HBsAg positive samples from our previous study that were of Minangkabau or Javanese ethnics collected from hospitals in western Java area (34 and 83 samples, respectively). These samples were later combined with the samples from Padang to further compared HBV genotype and subgenotype distribution between Minangkabau and Javanese ethnic (Figure 3).

### HBV genotyping

HBV DNA was extracted from 200 μl plasma using the QIAamp DNA blood mini kit (Qiagen, Hilden, Germany) according to the manufacturer’s instructions, and 80 μl of eluted DNA was stored at −70°C until use. HBV genotyping was performed by Polymerase Chain Reaction using genotype specific primers as described by Naito et al. [29]. The PCR products were run in agarose 2% and visualized under UV light.

### HBV pre-S and BCP mutations analysis

Pre-S region was amplified by PCR using PCR Core System (Promega, Madison, WI, USA) and primers as previously described [15]. The PCR products were purified with Wizard® SV Gel and PCR Clean-Up System (Promega, Madison, WI, USA), directly sequenced employing an ABI 3130xl Genetic Analyzer (Applied Biosystems, Inc., Foster City, CA, USA) with the Big Dye Terminator V3.1 Cycle Sequencing kit (Applied Biosystems, Inc.) using appropriate primers. Pre-S amino acid sequences were aligned and compared between each group to detect the mutations. BCP region was amplified as previously described [12]. Retrieved sequenced were aligned with reference sequences from GenBank and analyzed for mutation.

### Phylogenetic analysis

The HBV subgenotype was determined by phylogenetic analysis of the pre-S (pre-S1/S2) or HBsAg (S) sequences. The Pre-S or S region was amplified with nested PCR as previously described [15,30]. Reference sequences of all previously described HBV genotypes and subgenotypes were retrieved from GenBank database together with all samples obtained in this study to construct phylogenetic trees based on partial pre-S or S region by the neighbor-joining method [31] using the maximum composite likelihood method to calculate evolutionary distances [32]. Bootstrap resampling was performed 1,000 times. All phylogenetic analyses were carried out using the MEGA5 software [33]. The genotypes and subgenotypes of the samples were determined based on their phylogenetic co-clustering with the reference sequences. The possibility of recombination in the pre-S or S sequences were assessed in samples showing ambiguous phylogenetic clustering using web-based genotyping resource (Genotyping tool, NCBI http://www.ncbi.nlm.nih.gov/projects/genotyping/formpage.cgi) and further confirmed using SimPlot v3.2 software.

HBV pre-S and S gene sequences were deposited in GenBank under accession number JQ029166-JQ029269; JQ428829-JQ429078 and JX196538-JX196592. BCP sequences were deposited in GenBank under accession number JX196400-JX196537.

### Statistical analysis

All statistical analyses were performed using SPSS 17.0 software for Windows (SPSS Inc., Chicago, IL, USA). Statistical significances were determined using Fisher’s exact test, chi-square test, and *t*-test analysis whenever appropriate. *P*<0.05 was considered significant.

## Competing interests

The authors declare that they have no competing interests.

## Authors’ contribution

Siburian MD performed the experiment, data analysis and prepared the manuscript; Utama A designed the study and prepared the manuscript; Dhenni R data analysis and prepared the manuscript; Arnelis, Fanany I, Intan MDB, Kurniasih TS, Andriani F, and Afadlal S performed the experiments; Julianto EB, Rasman WS, Zubir N, and Mathew G coordinated and provided the collection of human materials and involved in editing the manuscript. All authors read and approved the final manuscript.
